# IRE1/JNK Is the Leading UPR Pathway in 6-OHDA-Induced Degeneration of Differentiated SH-SY5Y Cells

**DOI:** 10.3390/ijms25147679

**Published:** 2024-07-12

**Authors:** Natalia Siwecka, Grzegorz Galita, Zuzanna Granek, Wojciech Wiese, Ireneusz Majsterek, Wioletta Rozpędek-Kamińska

**Affiliations:** Department of Clinical Chemistry and Biochemistry, Medical University of Lodz, Mazowiecka 5, 92-215 Lodz, Poland; natalia.siwecka@stud.umed.lodz.pl (N.S.); grzegorz.galita@umed.lodz.pl (G.G.); zuzanna.granek@stud.umed.lodz.pl (Z.G.); wojciech.wiese@stud.umed.lodz.pl (W.W.); ireneusz.majsterek@umed.lodz.pl (I.M.)

**Keywords:** IRE1, JNK, PERK, unfolded protein response, ER stress, Parkinson’s disease, 6-OHDA, neurodegeneration

## Abstract

Parkinson’s disease (PD) is a neurodegenerative disorder which affects dopaminergic neurons of the midbrain. Accumulation of α-synuclein or exposure to neurotoxins like 6-hydroxydopamine (6-OHDA) induces endoplasmic reticulum (ER) stress along with the unfolded protein response (UPR), which executes apoptosis via activation of PERK/CHOP or IRE1/JNK signaling. The present study aimed to determine which of these pathways is a major contributor to neurodegeneration in an 6-OHDA-induced in vitro model of PD. For this purpose, we have applied pharmacological PERK and JNK inhibitors (AMG44 and JNK V) in differentiated SH-SY5Y cells exposed to 6-OHDA. Inhibition of PERK and JNK significantly decreased genotoxicity and improved mitochondrial respiration, but only JNK inhibition significantly increased cell viability. Gene expression analysis revealed that the effect of JNK inhibition was dependent on a decrease in *MAPK10* and *XBP1* mRNA levels, whereas inhibition of either PERK or JNK significantly reduced the expression of *DDIT3* mRNA. Western blot has shown that JNK inhibition strongly induced the XBP1s protein, and inhibition of each pathway attenuated the phosphorylation of eIF2α and JNK, as well as the expression of CHOP. Collectively, our data suggests that targeting the IRE1/JNK pathway of the UPR is a more effective option for PD treatment as it simultaneously affects more than one pro-apoptotic pathway.

## 1. Introduction

Parkinson’s disease (PD) is the second most common neurodegenerative disorder after Alzheimer’s with a prevalence of 2–3% in the population ≥65 years of age, which is expected to double by 2030 [1]. Dopamine (DA) deficiency in the substantia nigra pars compacta (SNpc) is specific to PD, and it results in typical motor features such as tremor, rigidity, and bradykinesia, as well as non-motor symptoms including hyposmia, sleep disorders, depression, and constipation. The neuropathological hallmarks of PD are the intracellular inclusions of misfolded α-synuclein called Lewy bodies, as well as loss of DA neurons in the SNpc; this ultimately leads to synaptic dysfunction, neuroinflammation, mitochondria-mediated apoptosis, and progressive neuronal loss [2]. In familial PD, mutations in genes such as *SNCA*, *PRKN*, *PINK1*, *LRRK2*, or *GBA1* contribute to improper folding and accumulation of α-synuclein within the endoplasmic reticulum (ER) lumen, which evokes ER stress and activation of the unfolded protein response (UPR) signaling pathway [3]. UPR is mediated by the three sensors located at the ER membrane: protein kinase RNA-like endoplasmic reticulum kinase (PERK), inositol-requiring enzyme 1 (IRE1), and activating transcription factor 6 (ATF6). While ATF6-dependent signaling is primarily regarded as cytoprotective, it has been demonstrated that the other two axes of the UPR, PERK and IRE1, play an important role in PD pathogenesis by triggering apoptosis of DA neurons [4,5,6,7,8]. The hyperactivation of both of these pathways under prolonged ER stress conditions switches the pro-adaptive response into pro-apoptotic via upregulation of specific, pro-apoptotic genes [9]. However, it still remains unclear exactly which of these UPR pathways contributes to apoptosis in the course of PD.

Once ER stress is triggered, the binding immunoglobulin protein (BiP/GRP78) chaperones bind to misfolded proteins and dissociate from the three UPR sensors. The first of them, PERK, subsequently undergoes dimerization and autophosphorylation [10]. Activated p-PERK catalyzes the phosphorylation of the α subunit of the eukaryotic translational initiation factor 2α (eIF2α), which results in an arrest in global protein translation and, at the same time, selective translation of the activating transcription factor 4 (ATF4) [11]. Upon long-term ER stress, ATF4 induces pro-apoptotic genes like *DNA damage-inducible transcript 3* (*DDIT3*)*,* encoding C/EBP homologous protein (CHOP), *DNA damage-inducible protein 34 (GADD34*)*,* or the B-cell lymphoma 2 (Bcl-2) family members [12]. The evidence for PERK signaling activation in PD was provided by a number of preclinical studies [13,14], and increased p-PERK and p-eIF2α levels were detected in brain specimens from PD patients [14,15]. Several reports have also confirmed the involvement of CHOP in dopaminergic neurodegeneration [13,16].

As in the case of PERK, BiP, by dissociating from IRE1, induces dimerization and autophosphorylation of the kinase. The cytosolic domain of IRE1 shows an atypical endoribonuclease (RNAse) activity, responsible for the unconventional splicing of *X-box binding protein 1* (*XBP1*) mRNA. The activated, spliced form of XBP1 (XBP1s) exerts a cytoprotective response by upregulating target genes. Under chronic stress conditions, IRE1 triggers cell apoptosis via interaction with TNF receptor-associated factor 2 (TRAF2), which subsequently induces apoptosis signal-regulating kinase 1 (ASK1) and c-Jun N-terminal kinase (JNK) [9]. Activated JNK in turn regulates various Bcl-2 family members, in particular the pro-apoptotic factors Bcl-2-like protein 11 (BIM) and BH3 Interacting Domain Death Agonist (BID). Distinct pro-apoptotic proteins such as BAX, BAK, AIP1, and PTP1B can interact with IRE1 to facilitate its RNase activity and thus increase the *XBP1* mRNA splicing [17]. It has been therefore suggested that IRE1-induced XBP1 splicing plays a protective role in the pathogenesis of PD [18], whereas overexpression of IRE1/JNK promotes DA neuron loss, neuroinflammation, progression of the disease, and shorter lifespan [19].

Several mitochondrial neurotoxins are a well-known cause of selective degeneration of DA neurons. As these cells are particularly susceptible to mitochondrial dysfunction, exposure to neurotoxins inevitably leads to their apoptosis. The dopaminergic SH-SY5Y neuroblastoma cell line treated with 6-hydroxydopamine (6-OHDA) constitutes a standard experimental model of PD. 6-OHDA inhibits mitochondrial complex I, upregulates Bax, caspase-3, and -9, and induces apoptosis in a mitochondria-dependent manner [20]. It also impairs mitochondrial transport in both DA and non-DA axons and significantly upregulates the transcripts associated with the UPR. The increased levels of UPR markers like BiP, p-eIF2α, CHOP, p-IRE1, XBP1s, and p-c-Jun were evidenced in in vitro and in vivo models of PD submitted to 6-OHDA treatment [13,21,22].

The present study aimed to investigate which of the pro-apoptotic UPR pathways, PERK/CHOP or IRE1/JNK, is responsible for apoptosis in differentiated SH-SY5Y cells exposed to 6-OHDA insult. The pharmacological inhibition of PERK/CHOP and IRE1/JNK signaling in the mentioned model was achieved using the specific inhibitors, namely AMG44 and JNK V. Thorough understanding of the effects of each individual UPR signaling pathway on the molecular pathogenesis of PD can bring us closer to the development of new targeted therapies against this incurable disease.

## 2. Results

### 2.1. Differentiation of SH-SY5Y Cells

**Figure 1:**
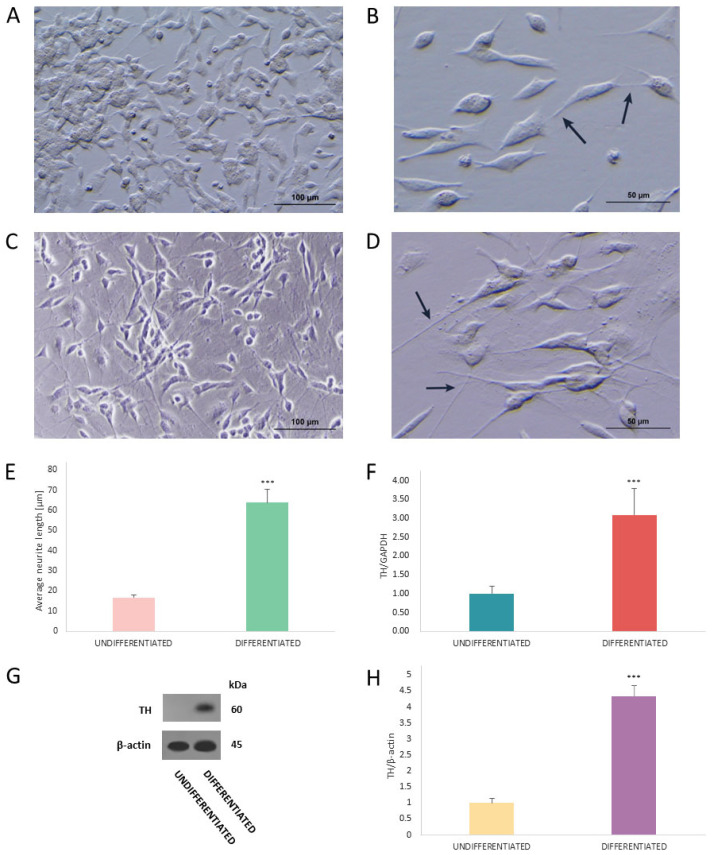
Morphological features of undifferentiated SH-SY5Y cells (**A**,**B**) and SH-SY5Y cells differentiated with retinoic acid (RA) (**C**,**D**). Neuron projections are indicated by arrows. Whilst undifferentiated cells are characterized by short processes and tend to grow in clusters, differentiated cells have significantly longer neurites and communicate with each other via neural network. The measurement of average neurite length (ANL) in undifferentiated and differentiated SH-SY5Y cells (**E**) showed a significant increase in ANL value for differentiated cells. The qRT-PCR analysis (**F**) revealed a significant upregulation of the expression of the *TH* gene encoding tyrosine hydroxylase (TH) in differentiated SH-SY5Y cells, whereas Western blot analysis (**G**,**H**) demonstrated a significant increase in TH protein expression in differentiated cells. The Student’s *t*-test was used in the statistical analysis. All the experiments were run in triplicate and the data are expressed as mean ± SD. *** *p* < 0.001 vs. undifferentiated.

Differentiation with retinoic acid (RA) was evaluated under the inverted microscope (Nikon, Tokyo, Japan). Upon RA treatment, there was an evident growth of neuronal processes and organization of cells into a neural network. Differentiation was evidenced by measuring the average neurite length (ANL) value. ANL in differentiated cells was over 4-fold higher than in undifferentiated cells (79 µm vs. 19 µm, respectively), and the difference was statistically significant. In addition, qRT-PCR and Western blot analyses revealed a significant increase in tyrosine hydroxylase (TH) expression, which is a marker of dopaminergic neurons, in differentiated SH-SY5Y cells (Figure 1).

### 2.2. The Cytotoxicity of 6-OHDA, PERK and JNK Inhibitors

**Figure 2:**
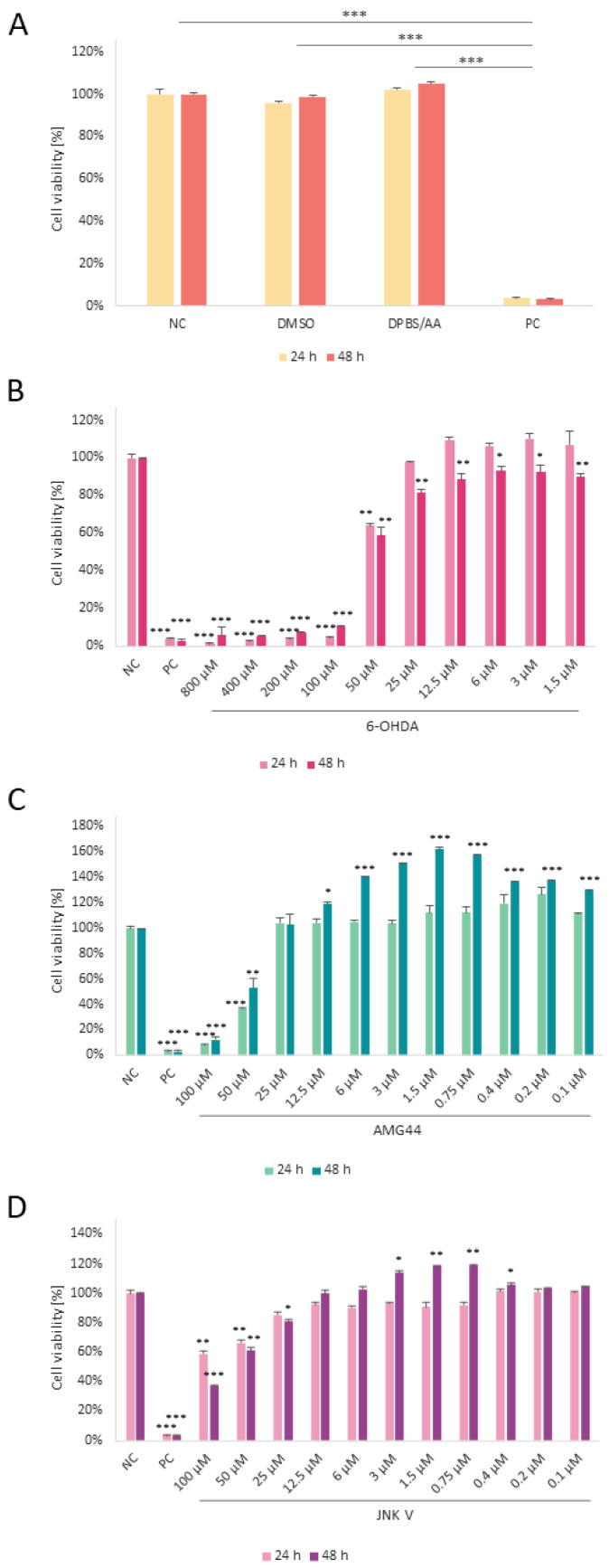
The cytotoxicity analysis of the used solvents (**A**), neurotoxin 6-hydroxydopamine (6-OHDA) (**B**), PERK inhibitor AMG44 (**C**), and JNK V inhibitor (**D**) in differentiated SH-SY5Y cells. Cells were incubated with the indicated compounds for 24 and 48 h. The one-way ANOVA with Bonferroni post hoc test was used in the statistical analysis. All the experiments were run in triplicate, and the data are expressed as mean ± SD, *** *p* < 0.001 for all groups (**A**), * *p* < 0.05, ** *p* < 0.01, *** *p* < 0.001 vs. negative control (**B**–**D**). Abbreviations: NC—negative control, untreated cells; DMSO—cells treated with the solvent, 0.1% dimethyl sulfoxide; DPBS/AA—cells treated with the solvent, 0.5% Dulbecco’s phosphate-buffered saline with 0.15% *w*/*v* ascorbic acid; PC—positive control, cells treated with 20% dimethyl sulfoxide.

The cytotoxicity of all used compounds was assessed by XTT assay. First, the cells were treated with the solvents of the tested substances, 0.1% DMSO and 0.5% DPBS with ascorbic acid (AA), which did not induce a significant cytotoxic effect in cells at any incubation time (Figure 2A). The cytotoxicity of 6-OHDA in differentiated SH-SY5Y neurons was evaluated by incubating cells with the neurotoxin for 24 and 48 h. The concentration range of 6-OHDA (800–1.5 µM) was selected based on the previous literature data. Due to our interest in long-term damage induced by 6-OHDA, the incubation time of 48 h was chosen for further experiments. In our study, the EC50 of 6-OHDA at 48 h incubation time amounted to 58 µM (Figure 2B). Based on the IC50 of both inhibitors described in the literature, we tested the AMG44 and JNK V compounds for potential toxicity in differentiated SH-SY5Y cells at 100–0.1 µM concentrations for 24 and 48 h. Cytotoxicity experiments revealed inhibition of cellular proliferation at relatively high concentrations of the inhibitors (≥50 μM) and a significant increase in cell proliferation at lower concentrations of both compounds, which is indicative of a typical hormetic effect (Figure 2C,D). Therefore, low concentrations of the indicated compounds (<12.5 μM) were selected for further experiments. 

### 2.3. Treatment with JNK Inhibitor Reduces 6-OHDA-Induced Toxicity

**Figure 3:**
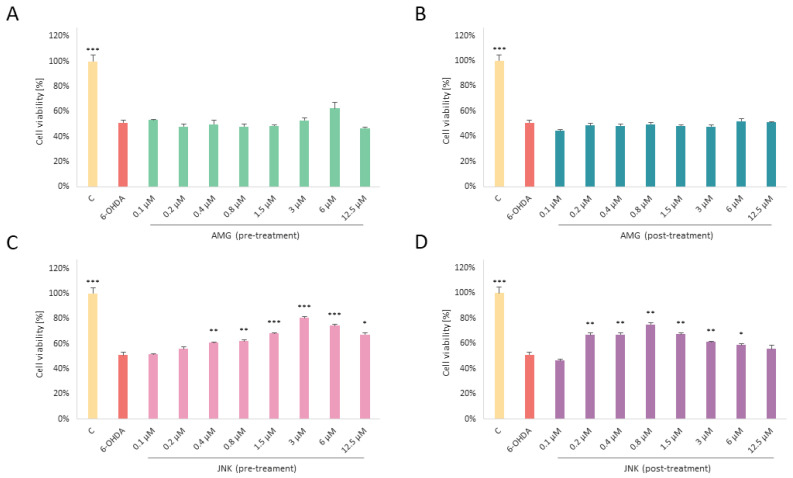
The effect of pre- (**A**) or post-treatment (**B**) with AMG44 and pre- (**C**) or post-treatment (**D**) with JNK V on the viability of differentiated SH-SY5Y cells exposed to neurotoxin 6-hydroxydopamine (6-OHDA). The one-way ANOVA with Bonferroni post hoc test was used in the statistical analysis. All the experiments were run in triplicate and the data are expressed as mean ± SD. * *p* < 0.05, ** *p* < 0.01, *** *p* < 0.001 vs. 6-OHDA. Abbreviations: C—control, untreated cells; AMG—AMG 44; JNK—JNK V.

In the next step of the cytotoxicity experiments, we wanted to assess whether inhibition of PERK/CHOP or IRE1/JNK decreases 6-OHDA-induced damage. Cells treated with EC50 6-OHDA for 48 h were exposed to 1 h pre- or post-treatment with AMG44 and JNK V compounds at the range of 12.5–0.1 µM. To our surprise, as far as treatment with AMG44 did not significantly affect the cell viability, JNK V effectively increased cell survival upon 6-OHDA insult, either when it was applied as a pre-treatment or post-treatment. Interestingly, in the post-treatment, relatively low doses of JNK V (0.8–3 μM) protected the cells from 6-OHDA-induced toxicity more effectively than higher doses of the compound, which once again indicates a hormetic effect of the compound. Overall, it seems that selective inhibition of JNK, but not PERK, has a significant effect on cell survival in 6-OHDA toxicity conditions (Figure 3). Based on the results of this assay, the most effective concentrations of the JNK V compound were chosen for further experiments (3 μM JNK V as a pre-treatment and 0.8 μM as a post-treatment). For the AMG44 inhibitor, the concentration of 6 μM was chosen as it slightly increased the cell viability in the pre-treatment (*p* = 0.051). At the same time, 6 μM was the highest non-toxic concentration of the inhibitor which increased cell viability in differentiated SH-SY5Y cells with a very high level of significance (*p* < 0.01, Figure 2C).

### 2.4. Inhibition of PERK and JNK Decreases 6-OHDA-Induced Genotoxicity

**Figure 4:**
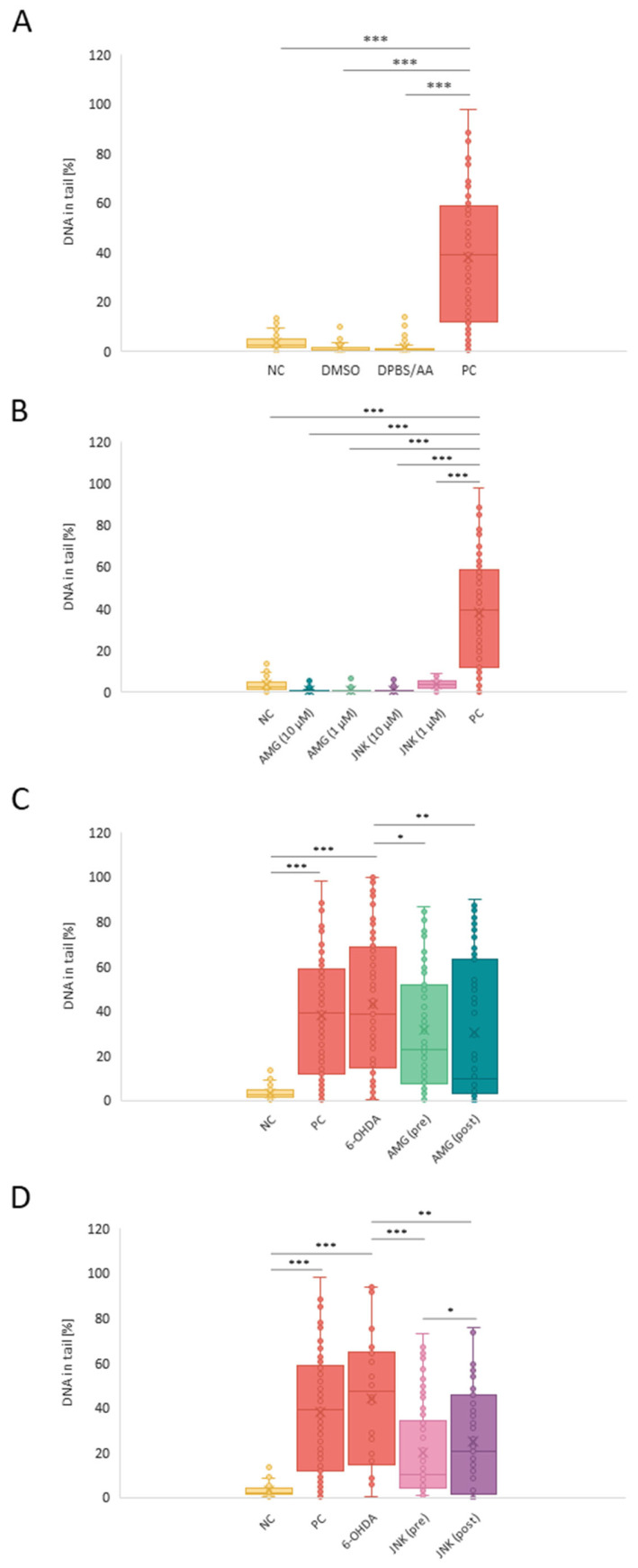
Evaluation of genotoxicity of the used solvents (**A**), PERK and JNK inhibitors AMG44 and JNK V (**B**) in differentiated SH-SY5Y cells, and the effect of treatment with AMG44 (**C**) or JNK V (**D**) on the level of DNA damage in differentiated SH-SY5Y cells exposed to 6-hydroxydopamine (6-OHDA). The Kruskal–Wallis test was applied for the statistical analysis. All the experiments were run in triplicate, and the data are expressed as median and interquartile range. * *p* < 0.05, ** *p* < 0.01, *** *p* < 0.001 for all groups. Abbreviations: NC—negative control, untreated cells; DMSO—cells treated with the solvent, 0.1% dimethyl sulfoxide; DPBS/AA—cells treated with the solvent, 0.5% Dulbecco’s phosphate-buffered saline with 0.15% *w*/*v* ascorbic acid; PC—positive control, cells treated with 20% dimethyl sulfoxide; AMG—AMG 44; JNK—JNK V; (pre)—pre-treatment; (post)—post-treatment.

The alkaline version of the comet assay (ACA) was applied to evaluate the level of DNA damage in cells exposed to 6-OHDA and treated with the AMG44 and JNK V compounds prior to and after the neurotoxic damage. Additional controls with cells exposed only to the solvents or the inhibitors (AMG44 and JNK V at 10 and 1 μM) for 48 h were also performed, and they did not demonstrate any genotoxicity of the tested compounds (Figure 4A,B). 6-OHDA exposure induced a significant genotoxic effect in treated cells (median 38.81% of damage), comparable to that of the positive control. To our surprise, pre-treatment with JNK V drastically decreased the 6-OHDA-induced DNA damage, with a median of 11.1%. Post-treatment with the compound was less effective in reducing 6-OHDA genotoxicity (26.92%), but the difference was still statistically significant. On the other hand, AMG44 pre-treatment significantly reduced 6-OHDA damage, but to a lesser extent than JNK V (24.57%), and the post-treatment with AMG44 was slightly more effective than pre-treatment (19.34%) (Figure 4C,D).

### 2.5. Pretreatment with PERK and JNK Inhibitors Increases the Oxygen Consumption Rate in Cells Exposed to 6-OHDA

**Figure 5:**
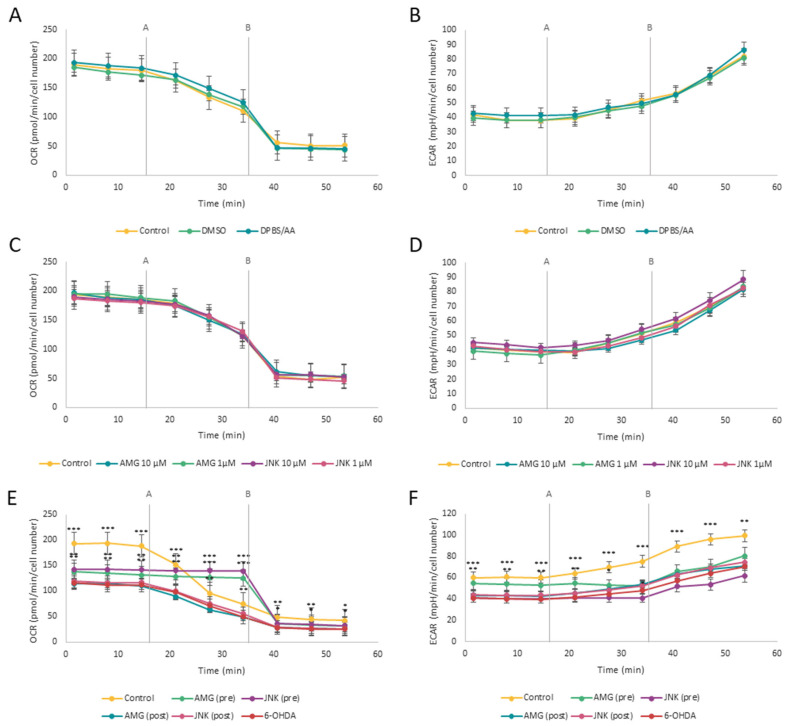
Evaluation of oxidative phosphorylation (**A**,**C**,**E**) and glycolysis (**B**,**D**,**F**) in real-time by Seahorse ATP Rate assay in differentiated SH-SY5Y cells treated with the solvents (**A**,**B**), AMG 44 and JNK V inhibitors (**C**,**D**), or treated with the inhibitors upon 6-hydroxydopamine (6-OHDA)-induced damage (**E**,**F**). The two-way ANOVA with Bonferroni post hoc test was used in the statistical analysis. All the experiments were run in triplicate, and the data are expressed as mean ± SD. * *p* < 0.05, ** *p* < 0.01, *** *p* < 0.001 vs. 6-OHDA (**E**,**F**). Abbreviations: control—untreated cells; DMSO—cells treated with the solvent, 0.1% dimethyl sulfoxide; DPBS/AA—cells treated with the solvent, 0.5% Dulbecco’s phosphate-buffered saline with 0.15% *w*/*v* ascorbic acid; AMG—AMG 44; JNK—JNK V; (pre)—pre-treatment; (post)—post-treatment.

Seahorse ATP Rate Assay is a very sensitive method for the real-time monitoring of mitochondrial respiration (oxidative phosphorylation) and acidification rate (glycolysis) in living cells by measuring OCR and ECAR, respectively. First, we examined whether tested inhibitors and solvents affect cellular respiration in differentiated SH-SY5Y cells. The OCR and ECAR values of cells treated with the compounds’ solvents (Figure 5A,B) or with AMG44 (1–10 μM) and JNK V (1–10 μM) did not significantly differ from the control cells without any treatment (Figure 5C,D). Cells exposed to 6-OHDA demonstrated a significant decrease in OCR (almost by half). This is not surprising considering the fact that 6-OHDA is a mitochondrial neurotoxin, known to inhibit complexes I and IV of the electron transport chain [23]. In 6-OHDA toxicity conditions, pre-treatment with either AMG44 or JNK V significantly increased the OCR values (both basal and after oligomycin injection), which at some points even exceeded the OCR in control cells. By contrast, post-treatment with each compound had no effect on oxidative phosphorylation in real-time measurement as compared to cells treated with only 6-OHDA. In addition, pre-treatment with AMG44 increased the basal ECAR in cells exposed to 6-OHDA (Figure 5E,F). It therefore seems like PERK and JNK inhibition desensitizes differentiated SH-SY5Y cells to oligomycin-induced damage and improves oxidative phosphorylation under 6-OHDA toxicity conditions. 

### 2.6. PERK Inhibition Decreases the Caspase-3 Level in 6-OHDA-Treated Cells

**Figure 6:**
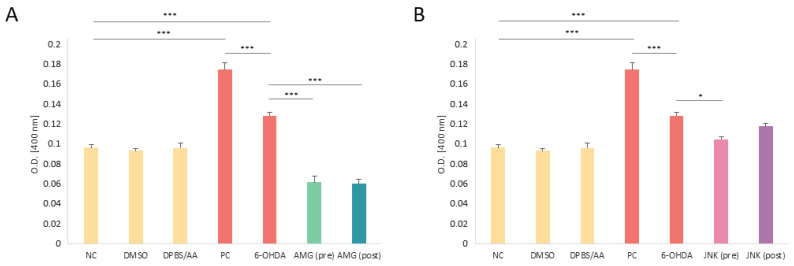
Colorimetric assessment of the level of caspase-3 in differentiated SH-SY5Y cells exposed to the solvents or 6-hydroxydopamine (6-OHDA) and treated with AMG44 (**A**) and JNK V (**B**) inhibitors upon 6-OHDA exposure. The two-way ANOVA with Bonferroni post hoc test was used in the statistical analysis. All the experiments were run in triplicate and the data are expressed as mean ± SD. * *p* < 0.05, *** *p* < 0.001 for all groups. Abbreviations: NC—negative control, untreated cells; DMSO—cells treated with the solvent, 0.1% dimethyl sulfoxide; DPBS/AA—cells treated with the solvent, 0.5% Dulbecco’s phosphate-buffered saline with 0.15% *w*/*v* ascorbic acid; PC—positive control, cells treated with 10 μM staurosporine; AMG—AMG 44; JNK—JNK V; (pre)—pre-treatment; (post)—post-treatment.

The apoptosis activation estimated by the caspase-3 level was performed in cells with 6-OHDA-induced damage and exposed to PERK and JNK inhibitors prior to and after 6-OHDA treatment. As expected, treatment with 6-OHDA resulted in a significant increase in caspase-3 activity. Surprisingly, both pre- and post-treatment with PERK inhibitor AMG44 significantly decreased the caspase-3 level, even below the level of the negative control, whereas JNK inhibitor had little significant effect on caspase-3 processing when applied as a pre-treatment (Figure 6). We have therefore concluded that the PERK inhibition may in fact decrease the level of caspase-3 upon 6-OHDA insult, but without any implications on cell survival, whereas the neuroprotective effect of JNK V is not completely dependent on caspase-3-processing.

### 2.7. PERK and JNK Inhibition Differently Affects the Expression of ER Stress-Related and Pro-Apoptotic Genes

**Figure 7:**
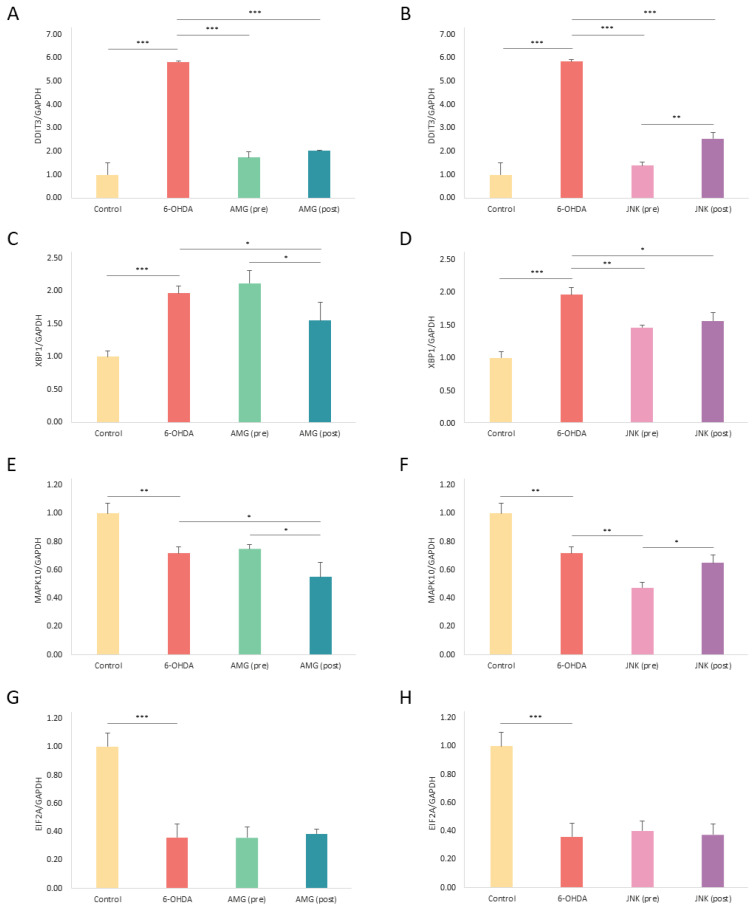
The mRNA expression levels of *DDIT3* (**A**,**B**), *XBP1* (**C**,**D**), *MAPK10* (**E**,**F**), and *EIF2A* (**G**,**H**) genes in differentiated SH-SY5Y cells exposed to 6-OHDA and treated with AMG44 (**A**,**C**,**E**,**G**) or JNK V (**B**,**D**,**F**,**H**) inhibitors. *GAPDH* was used as a reference gene. The two-way ANOVA with Bonferroni post hoc test was applied for the statistical analysis. All the experiments were run in triplicate and the data are expressed as mean ± SD. * *p* < 0.05, ** *p* < 0.01, *** *p* < 0.001 for all groups. Abbreviations: control—untreated cells; AMG—AMG 44; JNK—JNK V; (pre)—pre-treatment; (post)—post-treatment.

The gene expression analysis revealed a ~6-fold increase in the expression of *DDIT3* and a ~2-fold increase in the expression of total *XBP1* mRNA upon 48 h 6-OHDA exposure. At the same time, the expression levels of *MAPK10* and *EIF2A* were significantly decreased after 6-OHDA-induced damage. As these results did not correspond with the WB analysis, which revealed upregulation of p-JNK and p-eIF2α by 6-OHDA, we speculated that the accumulation or degradation of these factors was dependent on the post-transcriptional regulation. Strikingly, both pre- and post-treatment with the compounds AMG44 and JNK V vastly decreased the *DDIT3* expression level (Figure 7A,B). Treatment with JNK V also downregulated the expression of *XBP1* and *MAPK10* genes associated with the IRE1 pathway, whereas 1 h post-treatment with AMG44 induced slight downregulation of mentioned genes, which suggests that inhibition of the IRE1/XBP1/JNK branch could be an early event induced by the PERK inhibitor (Figure 7C–F). The expression of *EIF2A* remained unaffected by the inhibitors compared to the 6-OHDA-only treatment group (Figure 7G,H).

### 2.8. The Effect of PERK and JNK Inhibition on the Level of ER Stress-Related and Pro-Apoptotic Proteins

**Figure 8:**
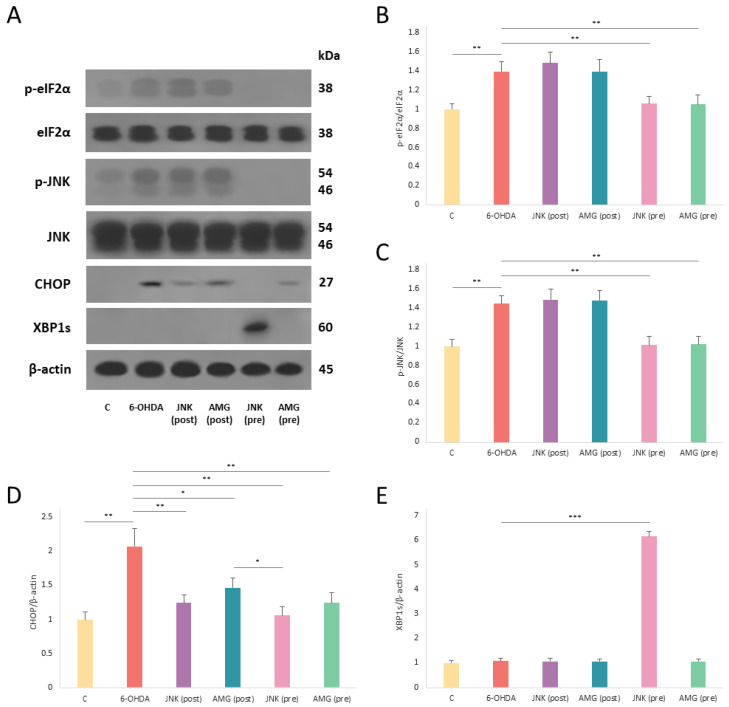
Western blot analysis of the expression level of the endoplasmic reticulum (ER) stress-related proteins (**A**) p-eIF2α, eIF2α (**B**), p-JNK, JNK (**C**), CHOP (**D**), and XBP1s (**E**), in differentiated SH-SY5Y cells exposed to 6-hydroxydopamine (6-OHDA) and treated with the AMG44 and JNK V compounds. β-actin was applied as a loading control. The two-way ANOVA with Bonferroni post hoc test was used in the statistical analysis. All the experiments were run in triplicate, and the data are expressed as mean ± SD. * *p* < 0.05, ** *p* < 0.01, *** *p* < 0.001 for all groups. Abbreviations: C—control, untreated cells; AMG—AMG 44; JNK—JNK V; (pre)—pre-treatment; (post)—post-treatment.

Western blotting demonstrated significant upregulation of p-eIF2α, p-JNK, and CHOP levels following 6-OHDA treatment (Figure 8A). The levels of p-eIF2α and p-JNK were effectively reduced by pre-treatment with PERK and JNK inhibitors, whereas post-treatment had no effect on the phosphorylation of these proteins (Figure 8B,C). Interestingly, the expression of pro-apoptotic CHOP factor was significantly downregulated by both pre- and post-treatment with JNK V and AMG44 (Figure 8D). Moreover, JNK inhibition was more effective at attenuating CHOP level than inhibition of PERK, which is regarded as the major regulator of CHOP. In addition, pre-treatment with JNK V inhibitor strongly induced the expression of XBP1s protein, which is known for its cytoprotective properties (Figure 8E). Thus, inhibition of JNK seems to exhibit more pleiotropic effect than PERK inhibition, as it affects the two potent pro-apoptotic factors and more than one UPR pathway at once.

## 3. Discussion

In our study, selective JNK inhibition provided neuroprotection against 6-OHDA toxicity more effectively than PERK inhibition. Specifically, JNK inhibition was more effective in terms of cytotoxicity, genotoxicity, and modulating expression of UPR-related genes and proteins. Surprisingly, the mechanism of action of the JNK inhibitor did not involve substantial suppression of caspase-3, which, besides JNK, could have been upregulated by the other pathways (e.g., the extrinsic pathway involving death receptors) [24]. Another possible explanation is that JNK may induce apoptosis in the mechanism bypassing the caspase-3 pathway, via activation of transcription factors c-Jun, p53, and p73, and this effect could be suppressed by JNK inhibition [25]. By contrast, PERK inhibition markedly decreased caspase-3 activity; however, this effect did not translate into increased survival of differentiated SH-SY5Y cells. Therefore, the caspase-3, although activated, might not be the leading inducer of apoptosis in the 6-OHDA model. In another study, CHOP has been pointed out as the central mediator of 6-OHDA-induced neurodegeneration, as its mRNA and protein expression levels were massively enhanced upon 6-OHDA treatment [22]. Interestingly, in our study, inhibition of JNK was equally or even more effective at decreasing the expression level of *DDIT3* mRNA and CHOP protein as compared to direct inhibition of the PERK/CHOP axis. This is in line with the previous reports that JNK activity is required for the activation and expression of *DDIT3* promoter [26]. As other studies indicate that JNK is also a major factor implicated in 6-OHDA-mediated apoptosis [27], the combined effect of inhibition of the two pro-apoptotic proteins JNK and CHOP at once by JNK inhibitor could give an explanation for its strong neuroprotective properties. The other possible mechanism of neuroprotective action of JNK V is the induction of XBP1s, which occurred only in cells pre-treated with JNK inhibitor prior to 6-OHDA damage. XBP1s is known for its cytoprotective role—when induced, it increases the expression of target genes involved in protein quality control. This is in accordance with the other study, which also provided mechanistic evidence for switching of the IRE1 signaling towards *XBP1* unconventional splicing upon inhibition of the IRE1/JNK axis during ER stress [26]. The other possible mechanism of exerting protection by inhibiting PERK or JNK is the switch of UPR towards the cytoprotective ATF6 pathway; however, we did not manage to evidence the activation of ATF6 in the present study.

The protective effects of PERK/CHOP and IRE1/JNK inhibition in 6-OHDA and other models of PD have previously been reported. One such example is the study on PERK inhibitor GSK2606414 in 6-OHDA-treated mice. Oral administration of GSK2606414 blocked the PERK pathway, protected dopaminergic neurons against 6-OHDA, increased DA levels, and improved motor performance in animals, but at the same time it induced pancreatic toxicity [14]. In view of the fact that GSK inhibitors of PERK turned out to be in fact RIPK1 inhibitors, other compounds were suggested to be used for the selective blocking of PERK activity [28]. AMG44 is one such compound that, in contrast to GSKs, has not been reported to damage the pancreas. In our study, selective inhibition of PERK by AMG44 did not significantly affect cell survival, which suggests that the protective effect of GSK could have resulted from the different experimental model used (6-OHDA mice) or the inhibitor’s effect on the other pathways. The effect of an aminopyrimidine JNK inhibitor, SR-3306, was evaluated in the same rodent model of PD. In vivo administration of SR-3306 significantly reduced the loss of dopaminergic neurons and their striatal projections in 6-OHDA-injected mice by decreasing the level of c-Jun phosphorylation [29]. The other JNK inhibitory compound, SP600125, which is specific towards the JNK2 isoform, has been tested in an in vitro model of PD, in which it rescued over 60% of PC12 cells upon 6-OHDA exposure. Mechanistically, inhibition of JNK2 reduced the cytochrome c release, caspase-3 cleavage, and the active JNKs pool in the nucleus in contrast to JNK1 inhibition, which had no effect [27]. The SP600125 inhibitor was also found to rescue neurodegeneration in a mouse model of PD induced by injection with the neurotoxin 1-methyl-4-phenyl-1,2,3,6-tetrahydropyridine (MPTP). The compound decreased the level of p-c-Jun, prevented apoptosis of dopaminergic neurons, and restored DA levels [30]. On the other hand, an eIF2α inhibitor C16 exerted neuroprotection in mouse cortical and dopaminergic neurons exposed to neurotoxins MPP+ and 6-OHDA. A decrease in apoptotic cell death by C16 was associated with suppression of ATF4 activation, which is a downstream target of PERK [31].

Whilst the inhibitors used in the present study have not yet been tested in the context of neurodegeneration in PD, AMG44 treatment relieved ER stress and normalized BK channel physiology in the cellular model of multiple sclerosis [32], and JNK V was effective at reducing the negative effects of stroke in gerbils [33]. The effectiveness of these compounds has not been investigated in any model of PD until now. Selective targeting of PERK was chosen due to literature data on the detrimental impact of the entire PERK/eIF2α/ATF4/CHOP axis on the course of PD [34]. Moreover, there are a broad range of PERK inhibitors that have been tested in various experimental conditions, whereas inhibitors with a narrower range of action, targeting PERK downstream effectors like ATF4 or CHOP, have not yet been developed and are not as widely available as PERK inhibitors. Also, gene silencing studies revealed that genetic ablation of ATF4 has contradictory effects in different models of PD: in PC12 cells exposed to 6-OHDA or MPP+ [35], it enhanced cell death, whereas a separate study has demonstrated that ATF4-deficient dopaminergic neurons are resistant to cell death induced by 6-OHDA, MPP+, and α-synuclein preformed fibrils [31]. At the same time, global inhibition of all PERK-mediated effects, including the eIF2α/ATF4-induced upregulation of protective genes or induction of the antioxidant Nrf2 pathway, could somehow hinder the full neuroprotective potential of PERK/CHOP inhibition in this study. Therefore, as inhibition of PERK may act as a double-edged sword, direct targeting of CHOP, which is a purely pro-apoptotic protein, could be a better option for potential PD treatment. On the other hand, in terms of the IRE1/XBP1/JNK axis, we have decided not to inhibit this signaling at the level of IRE1 so as not to suppress the kinase’s essential activities like *XBP1* splicing or RIDD. Therefore, targeting of IRE1 downstream effector JNK with mainly pro-apoptotic activity seemed to have more favorable outcomes in this study.

A number of natural compounds have also been assessed for alleviating ER stress in 6-OHDA toxicity models. These compounds are known for their pleiotropic effects and convergence on many different pathways, but most of them have one common feature—they exert neuroprotection by decreasing the level of UPR markers in various experimental models. In cellular or animal models of 6-OHDA toxicity, natural substances such as echinacoside, luteolin, cocoa extract, or β-asarone were found to, besides other mechanisms of action, inhibit the expression of the GRP78/PERK/eIF2α/ATF4/CHOP axis [36,37,38,39,40]. Other compounds like sulforaphane or ergothioneine demonstrated in vitro downregulation of CHOP as a neuroprotective mechanism [41,42]. Interestingly, low doses of oleuropein and rutin exerted protection in the 6-OHDA cellular model by downregulating the GRP78/PERK/CHOP pathway and XBP1 splicing, but they simultaneously upregulated IRE1 and ATF4 [43]. On the other hand, isoliquiritigenin, osthole, betanin, anandamide, carnosic acid, narcissoside, hydroxysafflor yellow A, and ginger root ethanol extract all demonstrated neuroprotective effects against 6-OHDA damage by suppressing phosphorylation of JNK [44,45,46,47,48,49,50,51].

Two FDA-approved drugs, adaptaquin and apomorphine, were shown to additionally inhibit ATF4/CHOP and p-JNK, respectively, in preclinical studies on 6-OHDA toxicity [52,53]. As apomorphine is an anti-parkinsonian drug, specifically a dopamine D1/D2 receptor agonist, this additional activity at the molecular level is highly desirable in PD therapy. Prosaposin-derived 18-mer peptide (PS18) reduced neurodegeneration in cellular and animal models of PD by decreasing the expression of the two UPR pathways, PERK/CHOP and ATF6 [54], whereas other proteins and peptides like SH3BP5, carnosine, albumin, or erythropoietin were shown to specifically inhibit JNK activity and thus exert protection against 6-OHDA [55,56,57,58]. Interestingly, in a recent study, it was found that cytisine, which is a partial α4β2 nicotinic acetylcholine receptor agonist, attenuated 6-OHDA-induced degeneration of dopaminergic neurons specifically in female mice. This phenomenon was linked to the combined effect of cytisine with 17-β-estradiol, as both compounds synergistically inhibited all three UPR pathways (ATF6, IRE1, and PERK) [59]. Altogether, all mentioned data indicate the involvement of UPR-dependent signaling pathways in 6-OHDA-induced neurodegeneration in PD.

6-OHDA was used in the present study for its documented involvement in PD pathology and ability to trigger neurodegeneration in various ways like oxidative stress, cytotoxicity, and mitochondrial insult. According to the literature, one of the mechanisms of 6-OHDA toxicity is the induction of ER stress conditions with upregulation of UPR markers, which is also the case for our study. As 6-OHDA is a neurotoxic agent endogenously produced in the human brain in the course of PD [60], studies on 6-OHDA toxicity may have possible clinical implications. A strong advantage of the present study is the application of the post-treatment with the inhibitors, which is often omitted in the study methodology. While most studies rely on the pre-treatment with the tested substances, such an approach is regarded as prophylaxis rather than actual drug treatment, and thus the obtained results in most cases do not translate into clinical practice. Herein, we have shown that post-treatment with JNK V significantly increases cell survival and reduces DNA damage upon 6-OHDA exposure. Although post-treatment had no effect on the expression of most UPR-related proteins, it significantly affected the expression of UPR markers at the mRNA level, which could be explained by a relatively short duration of the exposure (1 h). Previous in vivo studies have also reported that JNK V crosses the blood–brain barrier and exerts neuroprotection in a stroke model [33]. In the case of AMG44, to date, there is no information on tissue distribution of the compound, and in vivo studies are required to assess it.

One limitation of the present study is that it was performed in vitro, with only one cellular model applied. Notably, the neurotoxin-based profile of the study does not perfectly reflect human pathology. In terms of silencing PERK/CHOP and IRE1/JNK signaling, pharmacological inhibition only was applied. Thus, the results obtained require validation in other experimental models, especially in genetic and in vivo conditions. Also, as PD affects not only SNpc but also other regions of the brain, further experiments should consider other types of neural and glial cells. The other limitation of the present study is the relatively short duration of treatments with the inhibitors (1 h), especially in terms of the post-treatment, with a long duration of neurotoxin treatment (48 h). Longer incubation time with 6-OHDA at a moderate toxic dose was chosen to mimic the chronic and progressive nature of human disease better. Despite this fact, post-treatment with the compounds also appeared to be effective to some extent, and we suspect that longer treatment time with the compounds could further enhance the observed biological effects.

## 4. Materials and Methods

### 4.1. Cell Culture

The study was conducted on the SH-SY5Y human neuroblastoma cell line (ATCC, Manassas, VA, USA). This lineage constitutes a simple, easy-to-maintain, and inexpensive in vitro model for PD research, in contrast to primary dopaminergic neurons which are difficult to obtain and maintain in culture. The other advantages of this cell line include human origin, the ability to produce catecholamines, and the possibility of differentiation. These cells have been widely used in the literature to study the molecular mechanisms underlying PD pathogenesis as well as novel pharmacological approaches to PD. The cells were cultured in DMEM:F-12 medium (ATCC, Manassas, VA, USA) supplemented with 10% FBS (ATCC, Manassas, VA, USA) and 1% penicillin-streptomycin (ScienCell, Carlsband, CA, USA) in the cell culture incubator at standard conditions (37 °C, 5% CO_2_, 95% humidity). The cell culture medium was changed every 2–3 days. The cell passage was performed every 7 days: briefly, the cells were rinsed with DPBS (ScienCell, Carlsband, CA, USA), detached with 0.25% Trypsin/EDTA solution (ScienCell, Carlsband, CA, USA), centrifuged, and split at a 1:5 ratio. For each experiment, the culture did not exceed the 15th passage.

### 4.2. Differentiation

SH-SY5Y cell line, which normally presents tumor phenotype, may be differentiated to resemble dopaminergic neurons. The differentiation can be achieved by culturing the cells with specific compounds such as RA and/or by coating the culture vessels with extracellular matrix (ECM) proteins. In the present study, to acquire the dopaminergic phenotype, the cells were differentiated with RA (Sigma-Aldrich, Saint Louis, MO, USA), according to previously established protocols [61,62,63]. Briefly, cells were seeded in culture vessels coated with poly-L-lysine at the proper density (250,000 cells per 6-well plate) and, after adhesion (24 h), they were treated with complete culture medium supplemented with 3% FBS and 10 µM RA. The medium change and RA treatment was continued on days 3 and 5. On day 7, the cells were evaluated for differentiation by detecting the expression of TH by qRT-PCR and Western blot (for details, see Section 4.10 Gene Expression Analysis and Section 4.11 Immunoblotting) and by measuring the ANL of 100 randomly selected cells under the inverted microscope (Nikon, Tokyo, Japan). The ANL was measured using the NIS-Elements Advanced Research software, version 5.42 (Nikon, Tokyo, Japan). Following the differentiation (day 7), the actual experiments were performed.

### 4.3. Neurotoxin Treatment

The 6-OHDA stock powder (Sigma-Aldrich, Saint Louis, MO, USA) was stored at −20 °C in the dark. The 6-OHDA solution was freshly prepared before each experiment by dissolving the compound in DPBS (ScienCell, Carlsband, CA, USA) containing 0.15% *w*/*v* AA (Stanlab, Lublin, Poland). Based on the preliminary cytotoxicity results, the EC50 values for 6-OHDA were calculated using Statistica 13.3 software (StatSoft, Tulsa, OK, USA) by nonlinear regression of a dose–response relationship. The EC50 concentration of 6-OHDA solution at 48 h incubation was used in further experiments.

### 4.4. The UPR Inhibitors

For this study, we have chosen the compound 44 from Amgen (AMG44). AMG44 is a potent PERK inhibitor (IC50 = 6 nM), exhibiting very high selectivity for PERK, >160-fold over the majority of widely used kinases. It also demonstrates good pharmacokinetic properties for in vivo use [64] as well as no induction of pancreatic toxicity [65]. In contrast to other known PERK inhibitors, AMG44 does not interact with the receptor-interacting serine/threonine-protein kinase 1 (RIPK1), and thus it is regarded as a useful tool for selective pharmacological PERK inhibition [28]. To selectively inhibit the other UPR pathway, IRE1/JNK, we have used the JNK V inhibitor (other name: AS601245), which is a potent, cell-permeable, reversible ATP-competitive JNK inhibitor (hJNK1: IC50 = 150 nM, hJNK2: IC50 = 220 nM, and hJNK3: IC50 = 70 nM). It presents a 10- to 100-fold greater selectivity for JNK over commonly studied kinases. Its high potency for the JNK3 isoform especially makes it a great therapeutic candidate for central nervous system (CNS) diseases. It displays anti-inflammatory properties, and its in vivo efficacy has previously been demonstrated in mice, rats, and gerbils via oral, i.v., or i.p. administration [66]. Both inhibitors were purchased from Sigma-Aldrich, Saint Louis, MO, USA. The compounds were reconstituted in DMSO (BioShop Canada, Burlington, ON, Canada), and the stock solutions were kept in the dark at −20 °C.

### 4.5. Cytotoxicity Measurement

The XTT colorimetric assay (Invitrogen, Waltham, MA, USA) was applied to evaluate the toxicity of the compounds, cell proliferation, and viability. The assay is based on the conversion of the XTT tetrazolium salt into orange formazan product by metabolically active cells, and the intensity of the absorbance measurement indicates the number of living cells. First, the toxicity of 6-OHDA was measured after 24 h and 48 h exposure and the EC50 value was calculated. In order to exclude the potential cytotoxic effect of the applied inhibitors and solvents, the cells were also treated with AMG44 and JNK V at various concentrations, as well as 0.1% DMSO and 0.5% DPBS/AA (24 h and 48 h). Then, to assess if inhibition of PERK/CHOP and IRE1/JNK increases cell survival, the inhibitors were added either as a pre-treatment (1 h) or post-treatment (1 h) to cells exposed to 6-OHDA at EC50 (48 h). For each experiment, SH-SY5Y cells were seeded in 96-well plates coated with poly-L-lysine and differentiated as described above. Next, the cells were treated with the compounds for the indicated time period. Following the treatment, the medium was changed and the XTT/PMS mixture (25 μL) was added to each well and mixed. The cells were left for the next 3 h in the cell culture incubator. After incubation, the absorbance was measured at 450 and 630 nm using the Synergy HT Microplate Reader (BioTek Instruments, Winooski, VT, USA).

### 4.6. Treatment with the Compounds

In each experiment, the neurodegeneration of differentiated SH-SY5Y cells was induced by exposure to 6-OHDA at EC50 (58 µM) for 48 h. The inhibitors’ concentrations applied as a pre-treatment and post-treatment were chosen based on the cytotoxicity experiment results. Pre-treatment with the compounds (AMG44 at 6 μM or JNK V at 3 μM) was conducted for 1 h before 6-OHDA insult, and for post-treatment (AMG44 at 6 μM or JNK V at 0.8 μM), 1 h after 6-OHDA insult. 

### 4.7. Genotoxicity Assessment

To assess the effect of PERK and JNK inhibition against 6-OHDA-induced genotoxicity, the comet assay (alkaline version) was performed. The genotoxic effect was induced by exposing cells to 6-OHDA at EC50 for 48 h. Cells were treated with the UPR inhibitors as described above. Negative control constituted untreated cells, and positive control cells were treated with 20% DMSO for 1 h prior to the test. Additionally, the potential genotoxic effect of the applied solvents and inhibitors alone was also evaluated. After incubation with the compounds, the cells were dissociated with Trypsin/EDTA solution and centrifuged. The cell pellet was then resuspended in 100 μL of 0.37% LMP agarose (Sigma-Aldrich, Saint Louis, MO, USA) and placed on the microscope slides pre-coated with 0.5% NMP agarose. The resultant gel was solidified on ice for 10 min. The preparations were then lysed for 1 h at 4 °C in a lysis buffer (pH 10; 2.5 M NaCl, 10 mM Tris, 100 mM EDTA, 1% TritonX-100) and denatured for 20 min in a development buffer (pH 13; 300 mM NaOH, 1 mM EDTA). After incubation, the electrophoresis was performed for 20 min at 4 °C in the electrophoretic buffer (30 mM NaOH, 1 mM EDTA). Following this, the samples were rinsed thrice with distilled water and left to dry at RT. Finally, the preparations were stained with DAPI and analyzed under a fluorescent microscope (Nikon, Tokyo, Japan). The DNA damage in cells was determined based on the percentage of DNA in the comet tail in 100 randomly selected cells, and the measurement was performed using NIS-Elements Advanced Research software, version 5.42 (Nikon, Tokyo, Japan).

### 4.8. Seahorse ATP Rate Assay

In order to study the effect of PERK and JNK inhibition on cellular respiration (more specifically, oxidative phosphorylation and glycolysis) in 6-OHDA toxicity conditions, the Seahorse XFp Real-Time ATP Rate Assay (Agilent Technologies, Santa Clara, CA, USA) was applied. This test estimates the cellular metabolic activity by measuring the oxygen consumption rate (OCR) and extracellular acidification rate (ECAR). The cells were seeded in the Seahorse XF HS Miniplates (Agilent Technologies, Santa Clara, CA, USA), differentiated, and treated with the compounds as described above. Untreated cells served as control. The day before the assay, the Seahorse XFp Sensor Cartridge was hydrated overnight in a non-CO_2_ incubator as described in the manufacturer’s instructions. On the day of assay, the cartridge was rehydrated with the Seahorse XF Calibrant (Agilent Technologies, Santa Clara, CA, USA) and incubated for the next 45 min. The culture medium was removed from each well and the cells were washed with the prewarmed assay medium (pH 7.4) containing Seahorse XF DMEM, 10 mM glucose, 1 mM sodium pyruvate, and 2 mM L-Glutamine (Agilent Technologies, Santa Clara, CA, USA) according to the manufacturer’s protocol. Then, cells were incubated at 37 °C in a non-CO_2_ incubator for 45 min to let the medium achieve the proper temperature and pH before measurement. The ATP synthase inhibitor oligomycin (2.5 μM) and the mitochondrial complex I/III inhibitors rotenone/antimycin A (0.5 μM) solutions were prepared and loaded onto appropriate cartridge ports as described in the manufacturer’s protocol. The analysis was performed by means of Seahorse XF HS Mini Analyzer (Agilent Technologies, Santa Clara, CA, USA). Three baseline measurements were taken initially, and another three measurements were taken after injection of each compound.

### 4.9. Apoptosis Evaluation

The Caspase-3 colorimetric assay (Abcam, Cambridge, UK) was used to determine the effect of PERK and JNK inhibition on 6-OHDA-induced apoptosis. The assay protocol is based on the cleavage of the labeled substrate DEVD-pNA with a formation of the chromophore p-nitroaniline (p-NA). Treatment with the compounds was performed as described above. The negative control constituted untreated cells, whereas positive control cells were treated with 10 μM staurosporine for 24 h. Following the incubation, the cells were dissociated with Trypsin/EDTA solution and centrifuged. The obtained pellet was resuspended in 50 μL of chilled Lysis Buffer, and the lysate was incubated on ice for 10 min. Then, the samples were centrifuged at 10,000× *g* for 1 min and the supernatant was transferred to fresh microcentrifuge tubes and put on ice. The protein concentration was then measured by Pierce™ BCA Protein Assay (Thermo Scientific, Waltham, MA, USA) and adjusted to 100 μg protein per sample. Each sample was loaded onto a microplate (50 μL/well). Then, the Caspase Reaction Mix containing 2X Reaction Buffer and DTT was prepared and 50 μL was added to each well. Subsequently, 5 μL of the substrate solution (4 mM DEVD-pNA) was added to each sample well and the background well. The microplate was incubated at 37 °C for 120 min. The amount of p-NA was quantified using the Synergy HT Microplate Reader (BioTek Instruments, Winooski, VT, USA) by reading the absorbance at 400 nm.

### 4.10. Gene Expression Analysis

To confirm cell differentiation, the mRNA expression level of the *TH* gene was detected, and then the expression of selected genes related to IRE1 and PERK signaling pathways was assessed by qRT-PCR analysis in cells exposed to UPR inhibitors and 6-OHDA. Cell differentiation and compound treatments were performed as described above; control cells remained untreated. After incubation with the compounds, total RNA was isolated from cells by the PureLink™ RNA Mini Kit (Invitrogen, Waltham, MA, USA) according to the manufacturer’s instructions. The amount of RNA in the samples was quantified and normalized using the Synergy HT Microplate Reader (BioTek Instruments, Winooski, VT, USA). Then, the isolated RNA was transcribed into cDNA to a final concentration of 100 ng by the High-Capacity cDNA Reverse Transcription Kit (Applied Biosystems, Waltham, MA, USA) as described in the manufacturer’s protocol. The TaqMan™ Gene Expression Assays (Applied Biosystems, Waltham, MA, USA) were applied to analyze the expression profile of the *TH* gene or *MAPK10*, *XBP1*, *EIF2A*, and *DDIT3* genes. *GAPDH* served as a reference gene; the assays’ serial numbers are listed in Table 1. The total reaction volume was 20 μL, which included the following reagents: 1 μL cDNA, 1 μL probes, 10 μL TaqMan™ Universal PCR Master Mix II (Applied Biosystems, Waltham, MA, USA), and 8 μL nuclease free water (Invitrogen, Waltham, MA, USA). The PCR reaction was performed using the Bio-Rad CFX96 system (Bio-Rad, Hercules, CA, USA) in the following order: initial denaturation step (15 min, 95 °C), followed by the cycling—denaturation (10 s, 95 °C) and annealing/extension (60 s, 60 °C), 40 cycles each. The quantification of the obtained data was based on the calculation of 2^−∆∆Ct^ values.

### 4.11. Immunoblotting

Western blot analysis was performed to confirm the differentiation of SH-SY5Y cells and then to determine the effect of PERK and JNK inhibition on the expression of the selected UPR-associated proteins in cells exposed to 6-OHDA. Cells were differentiated and treated with the compounds as described above, except from the control cells that remained untreated. Then, the cells were harvested and the total protein was extracted using Minute™ Total Protein Extraction Kit (Invent Biotechnologies, Plymouth, MN, USA). The protein amount in the samples was measured and normalized based on the Pierce™ BCA Protein Assay (Thermo Scientific, Waltham, MA, USA). The protein samples were subsequently denatured at 70 °C for 10 min and loaded onto gel wells and then underwent electrophoresis for 50 min using the NuPage™/XCell SureLock™ system (Invitrogen, Waltham, MA, USA). Then, the proteins were transferred from the gel onto a PVDF membrane (Invitrogen, Waltham, MA, USA) for 1 h, and the membrane was blocked for the next 1 h in 5% BSA or skim milk (BioShop Canada, Burlington, ON, Canada) in 1X TBST (Thermo Scientific Chemicals, Waltham, MA, USA). BSA was used as a blocking agent for phosphoproteins, whereas skim milk was used for non-phosphoproteins. Next, the membranes were incubated overnight in the BSA/milk 1X TBST solution containing primary monoclonal antibodies against, depending on the experiment, TH (1:1000; Cell Signaling Technology, Danvers, MA, USA) or eIF2α, p-eIF2α, CHOP, XBP1s, JNK, and p-JNK (1:1000; Cell Signaling Technology, Danvers, MA, USA). β-Actin was applied as a loading control; all applied antibodies are listed in Table 2. On the next day, the membranes were washed thrice with 1X TBST and incubated with the secondary HRP-linked antibodies (1:5000; Cell Signaling Technology, Danvers, MA, USA). After the final wash in TBST, the membrane was incubated for 5 min in the dark with the SuperSignal™ West Pico Chemiluminescent Substrate (Thermo Scientific, Waltham, MA, USA), and the immune complexes were detected by the enhanced chemiluminescence (ECL) using the ChemiDoc™ Imaging System (Bio-Rad, Hercules, CA, USA). Densitometry was calculated using the NIS-Elements Advanced Research software (Nikon, Tokyo, Japan).

### 4.12. Statistical Analysis

The statistical analysis was performed using the Statistica 13.3 software (StatSoft, Tulsa, OK, USA). To determine whether the data have a normal distribution, the Shapiro–Wilk test was used, and the homogeneity of variance was evaluated by Levene’s test. In all cases except for the genotoxicity analysis, the data were homogenous and normally distributed, and therefore the comparison between the two groups was performed by the Student’s *t*-test, whereas comparisons between multiple groups were performed by one-way or two-way ANOVA with Bonferroni post hoc test. In the genotoxicity assay, the statistical analysis was conducted using the Kruskal–Wallis test. All the experiments were run in triplicate, which involved cells with the same passage number. The data in the graphs are expressed as mean ± SD (unless otherwise stated), and the statistically significant differences are displayed as follows: * *p* < 0.05, ** *p* < 0.01, *** *p* < 0.001.

## 5. Conclusions

Taken together, our data support the idea that IRE1/JNK is the leading pro-apoptotic pathway of the UPR in ER stress-mediated 6-OHDA toxicity in the cellular model of PD. Inhibition of JNK rescues cells from 6-OHDA-induced cytotoxicity, genotoxicity, and attenuation of mitochondrial respiration by decreasing the expression of UPR markers p-JNK, p-eIF2α, and CHOP, and by inducing the XBP1s protein. In addition, the effectiveness of the short-duration post-treatment of JNK V provides strong clinical implications for the potential use of JNK inhibitors in future therapeutic approaches against PD. Accumulating evidence indicates the ER stress-mediated apoptosis of dopaminergic neurons as the central molecular event responsible for neurodegeneration in PD. Further studies are needed in order to confirm the advantages of targeting JNK-mediated apoptosis in PD, which could one day provide the first disease-modifying strategy against PD.

## Figures and Tables

**Table 1:** The ID numbers of the applied TaqMan™ Gene Expression Assays.

Gene Name	Encoded Protein	Assay ID
*TH*	TH	Hs00165941_m1
*EIF2A*	eIF2α	Hs00230684_m1
*DDIT3*	CHOP	Hs01090850_m1
*XBP1*	XBP1	Hs00231936_m1
*MAPK10*	JNK3	Hs00959268_m1
*GAPDH*	GAPDH	Hs99999905_m1

**Table 2:** The catalog numbers of the applied primary antibodies obtained from the Cell Signaling Technology.

Antibody Name	Dilution	Catalog No.
Tyrosine Hydroxylase Antibody	1:1000	#2792
Phospho-eIF2α (Ser51) Antibody	1:1000	#9721
eIF2α Antibody	1:1000	#9722
CHOP (D46F1) Rabbit mAb	1:1000	#5554
XBP-1s (D2C1F) Rabbit mAb	1:1000	#12782
SAPK/JNK Antibody	1:1000	#9252
Phospho-SAPK/JNK (Thr183/Tyr185) Antibody	1:1000	#9251
β-Actin (13E5) Rabbit mAb	1:1000	#4970
Anti-rabbit IgG, HRP-linked Antibody	1:5000	#7074

## Data Availability

The data generated in the present study may be requested from the corresponding author.
